# Integrative network analysis revealed the molecular function of folic acid on immunological enhancement in a sheep model

**DOI:** 10.3389/fimmu.2022.913854

**Published:** 2022-08-12

**Authors:** Bing Wang, Heqiong Li, Zhen Li, Bo Wang, Han Zhang, Boyan Zhang, Hailing Luo

**Affiliations:** State Key Laboratory of Animal Nutrition, College of Animal Science and Technology, China Agricultural University, Beijing, China

**Keywords:** folic acid, metabolomics, transcriptomics, lipid metabolism, immune function

## Abstract

We previously observed the beneficial role of folic acid supplemented from maternal or offspring diet on lamb growth performance and immunity. Twenty-four Hu lambs from four groups (mother received folic acid or not, offspring received folic acid or not) were used in the current study, which was conducted consecutively to elucidate the molecular regulatory mechanisms of folic acid in lambs by analyzing blood metabolome, liver transcriptome, and muscle transcriptome. Serum metabolomics analysis showed that L-homocitrulline, hyodeoxycholic acid, 9-Hpode, palmitaldehyde, N-oleoyl glycine, hexadecanedioic acid, xylose, 1,7-dimethylxanthine, nicotinamide, acetyl-N-formyl-5-methoxykynurenamine, N6-succinyl adenosine, 11-cis-retinol, 18-hydroxycorticosterone, and 2-acetylfuran were down-regulated and methylisobutyrate was up-regulated by the feeding of folic acid from maternal and/or offspring diets. Meanwhile, folic acid increased the abundances of *S100A12* and *IRF6* but decreased *TMEM25* in the liver. In the muscle, *RBBP9, CALCR, PPP1R3D, UCP3, FBXL4, CMBL*, and *MTFR2* were up-regulated, *CYP26B1* and *MYH9* were down-regulated by the feeding of folic acid. The pathways of bile secretion, biosynthesis of unsaturated fatty acids, linoleic acid metabolism, and herpes simplex virus 1 infection were changed by folic acid in blood, liver, or muscle. Further integrated analysis revealed potential interactions among the liver, blood, and muscle, and the circulating metabolites, hub gene, and pathways, which might be the predominant acting targets of folic acid in animals. These findings provide fundamental information on the beneficial function of folic acid no matter from maternal or offspring, in regulating animal lipid metabolism and immune enhancement, providing a theoretical basis for the use of folic acid from the view of animal health care.

## Introduction

Folic acid (FA) is a water-soluble B vitamin involved in purine, pyrimidine and methionine metabolism as a methyl carrier (1), which plays a critical role in DNA methylation and methionine metabolism. Generally, the requirement of FA in ruminants can be met by producing the ruminal microflora (2). However, dietary supplements of FA higher than 0.5 mg/kg body weight improved animal performance and feed efficiency with increased serum folate concentrations and milk folate concentrations in most studies of ruminants (3, 4). We recently found that the supplementation of rumen-protected FA in the maternal and/or lamb diets could increase the growth performance of lambs (5) and improve slaughter performance and meat quality (6).

Furthermore, it was found that FA significantly affects nucleic acid synthesis, DNA repair, cell growth, differentiation, apoptosis, cancer prevention (7), muscle development (8), and energy metabolism (9). In addition, FA plays a crucial role in the healthy balance of the immune system and plays a double-edged sword role in offspring health *via* mediating DNA methylation (10, 11). Inadequate levels of FA can drastically alter immune responses (11). It was also found that maternal FA deficiency changed the methylation of genes and pathways associated with neurodevelopment and learning/memory abilities in male rat offspring (12). Similarly, FA supplementation could enhance immunity or anti-inflammation (8, 13) and stimulate hepatic gene expression in the IGF-1/PI3K/mTOR pathway (14) in ruminants.

Our previous studies showed that FA improved animal growth and meat quality (5, 6); in the meantime, it had significant effects on animal health-related characteristics in sheep, such as serum high-density lipoprotein cholesterol and very low-density lipoprotein (5) and serum immune globulins (8). FA supplementation could affect proliferation and apoptosis in bovine mammary epithelial cells by increasing the ratio of Bcl-2/Bax protein levels (15). However, few studies have investigated FA’s systematic molecular regulatory mechanism, especially for their function in immunity, in ruminant species. In addition, the gut-liver-muscle axis is associated with many diseases in patients (16, 17). Therefore, this study aimed to identify the FA’s biological function on immunological enhancement in a sheep model using multi-omics through the liver-muscle axis. We speculated that an integrative network of blood, liver, and muscle directly responds to FA’s maternal and/or offspring supplementation.

## Materials and methods

### Experimental design and sampling

The information of rumen-protected FA has been described previously (9). The present study was conducted at Jiangsu Qianbao Animal Husbandry Co. Ltd. For the experiment, 120 Hu ewes (24 ± 4.2 months of age and 44.6 ± 5.43 kg of body weight, mean ± SD) with two parity and showing signs of estrus (based on vaginal examination) were selected and then randomly allocated into three groups (forty ewes in each group). The experimental ewes received either 0 mg (M0), 16 mg (M16), or 32 mg (M32) FA as rumen-protected FA per kg DM from mating to lambing. The added dose of FA was according to a previous study in beef steers (18). The rumen-protected FA contained 2% FA, 60% hydrogenated fat (ratio of C16:0 to C18:0 = 2:1), 16% bentonite powder, and 22% calcium stearate (rumen-protected rate 92.6%). All newborn lambs were nursed and weaned at 50 d of age. Then the twenty-two offspring were selected from each maternal group and allocated into two groups based on litter size, sex, and bodyweight to receive either 0 mg (C) or 4.0 mg/kg (F) rumen-protected FA (DM in their diets). Altogether, 66 Hu lambs (50 ± 4.3 d of age and 16.4 ± 3.84 kg of body weight) were selected and divided into a total of six groups. All the lambs were raised separately in individual pens. The feeding experiment of the weaned lambs lasted for 130 d and included a 10-d adaptation period followed by a 120-d collection period. The detailed procedures for the ewes and lambs raising were shown in our published studies (5, 6). Six lambs from each group were selected randomly for slaughtering. Due to the significant beneficial roles of M0F (only offspring received 4 mg FA), M16C (only ewe received 16 mg FA), M16F (ewe received 16 mg FA and offspring received 4 mg FA) on animal growth and health properties from our previous studies (5, 6), we selected these three groups and M0C (no FA received) for further multi-omics analysis. Three comparisons among the four groups (M0C vs. M0F, M0C vs. M16C, M0C vs. M16F) were conducted in the current study to reveal the specific function of FA in lambs.

The blood samples on d 180 before morning feeding were collected from the jugular vein, and the serum was achieved by centrifuging the blood at 4°C for 10 min at 2000 × g. After slaughtering, longissimus dorsi and liver tissues (approximately 2 g) were collected and placed in cryopreserved tubes for further transcriptome analysis. All these samples were stored under liquid nitrogen.

### Metabolomics analysis

Serum sample extraction process: 1) The samples were thawed on ice. After thawing, vortex for 10 s to mix well; 2) Take 50 μL of the sample into an EP tube, add 150 μL of pre-cooled ice methanol (containing 1 μg/mL of 2-chlorophenylalanine as an internal standard); 3) Vortex for 3 min, 12,000 r/min, and centrifuge at 4°C for 10 min; 4) After centrifugation, pipette 200 μL of the supernatant into another new EP tube; 5) Centrifuge the supernatant with 12,000 r/min at 4°C for 5 min, and take 150 μL of the supernatant into the lining tube of the injection bottle for LC-MS/MS analysis.

The data acquisition instrument system mainly includes Ultra Performance Liquid Chromatography (UPLC) (Shim-pack UFLC SHIMADZU CBM30A, https://www.shimadzu.com/) and tandem mass spectrometry (MS/MS) (QTRAP^®^, https://sciex.com/). The liquid conditions mainly include Column: Waters ACQUITY UPLC HSS T3 C18 1.8 µm, 2.1 mm×100 mm; Mobile phase: ultrapure water (0.04% acetic acid) in phase A, acetonitrile (0.04% acetic acid) in phase B; Elution gradient: water/acetonitrile (95:5 V/V) at 0 min, 5:95 V/V at 11.0 min, 5:95 V/V at 12.0 min, 95:5 V/V at 12.1 min, 14.0 The min was 95:5 V/V; the flow rate was 0.4 mL/min; the column temperature was 40°C; the injection volume was 2 μL. The mass spectrometry conditions mainly include electrospray ion source temperature 500 °C, mass spectrometry voltage 5500 V (positive), -4500 V (negative), ion source gas I (GS I) 55 psi, gas II (GS II) 60 psi, curtain gas 25 psi with the Collision Induced Ionization parameter set to high. Each ion pair is scanned in triple quadrupole (Qtrap) according to the optimized declustering voltage and collision energy. Quality control samples prepared by mixing sample extracts were inserted into every 10 assay samples to monitor the repeatability of the analysis process. The high stability of the instrument provides an essential guarantee for the repeatability and reliability of the data.

Mass spectral data were processed using the software Analyst 1.6.3. The metabolites of the samples were qualitatively and quantitatively analyzed by mass spectrometry based on the local metabolic database. Before doing the difference analysis, the principal component analysis was first performed, and then the orthogonal partial least squares discriminant analysis (OPLS-DA) was conducted. Differential metabolites were screened by combining fold change and the variable importance in projection (VIP) value of the OPLS-DA model. Screening criteria: select metabolites with VIP ≥ 1 and fold change > 1.2. Differential metabolites were annotated and displayed using the Kyoto Encyclopedia of Genes and Genomes (KEGG, http://www.kegg.jp) database.

### Transcriptomics analysis

Total RNA from liver and muscle tissue was extracted using EASYspin fibrous tissue RNA rapid extraction kit (RN44, Aidlab Biotechnologies Co., Ltd, Beijing, China), and all steps were carried out in strict accordance with the kit instructions. After the sample was qualified, magnetic beads with Oligo (dT) were used to enrich the mRNA. Then AMPure XP beads were used to purify the double-stranded cDNA, and then perform end repair, add A, and add adapters to the purified double-stranded cDNA. AMPure XP beads selected the size of the double-stranded cDNA, and finally, PCR amplification was performed to construct a cDNA library. Before sequencing, the Agilent 2100 was used to detect the size of the inserted fragment of the library. The effective concentration of the library was accurately quantified by the qPCR method (the effective concentration of the library was > 4 n*M*). Finally, Illumina Hiseq4000, PE 150, was used for On-board sequencing (Beijing Aoweisen Gene Technology Co., Ltd).

The raw data (raw reads) obtained by sequencing had minor contamination, and low-quality reads. To prevent the impact of subsequent analysis, they were filtered through in-house Perl scripts and used Trimmomatic software (v0.33) to remove bands. Remove reads with N (indeterminate base) content greater than 10%; remove reads with low-quality bases (Q ≤ 20) content greater than 50%. Finally, the sequencing quality Q20 > 95%, Q30 > 89% (Q20, Q30: the ratio of the number of bases with Phred quality value greater than 20, 30 to the total number of bases (clean data)) were selected to do follow-up comparison and data analysis. Alignment analysis with the reference genome of Texel sheep Oar_v3.1 (http://ftp.ensembl.org/pub/release-92/fasta/ovis_aries/dna/) was performed by Tophat2 (v2.1.0) software. The raw reads of the liver and muscle transcriptome sequencing are available at NCBI SRA (PRJNA822866 and PRJNA822707).

HTSeq (v 0.5.4 p3) software was used to analyze the gene expression level of each sample, and the model was union. Fragments per kilobase of exon model per million mapped reads (FPKM) is the number of fragments per kilobase per million reads from a gene that is used to measure gene expression levels (19). Only genes with FPKM > 1 that reached the expression threshold were analyzed in subsequent analyses. Gene differential expression analysis was performed using DESeq (1.10.1) on Reading count data obtained from gene expression level analysis (20). The standard of differential expressed gene screening was *P* < 0.05. KEGG was used to study the enrichment of significant pathways involved in differentially expressed genes.

### qPCR validation

Total RNA from the liver and muscle was reverse transcribed to cDNA using the TRUEscript RT (+gDNA Eraser) cDNA Synthesis Kit (Code No. PC5401, Aidlab Biotechnologies Co., Ltd, Beijing, China). Quantitative real-time PCR (qRT-PCR) was performed with the SYBR Green I (2×SYBR qPCR Mix) kit (Code No. PC3302, Aidlab Biotechnologies Co., Ltd, Beijing, China) and a LineGene 9640 instrument (BIOER Technology Co., Ltd, Hangzhou, China). The PCR conditions were set as follows: 1 cycle at 95°C for 3 min, 40 cycles of 95°C for 10 s and 60°C for 30 s, followed by a melting curve programme (60 to 95°C). The primers targeting hepatic *IRF6*, and muscular *FBXL4, UCP3*, and *CYP26B1*were listed in **Table S1**. Actin beta (*ACTB*) was used as a reference gene. The relative mRNA abundance of each gene was analyzed using the 2^-ΔCT^, ΔCT = CT_target mRNA_ – CT_house keeping mRNA_. Only M0C and M16F groups were used in the qPCR validation.

### Correlation analysis

Spearman correlation coefficients (r) were calculated in the R program, and *P* < 0.01 was considered statistically significant. The network file was visualized using Cytoscape (v3.8.0) software to present the core and hub gene biological interactions.

## Results

### Serum metabolomics and metabolic pathways

**Figure 1:**
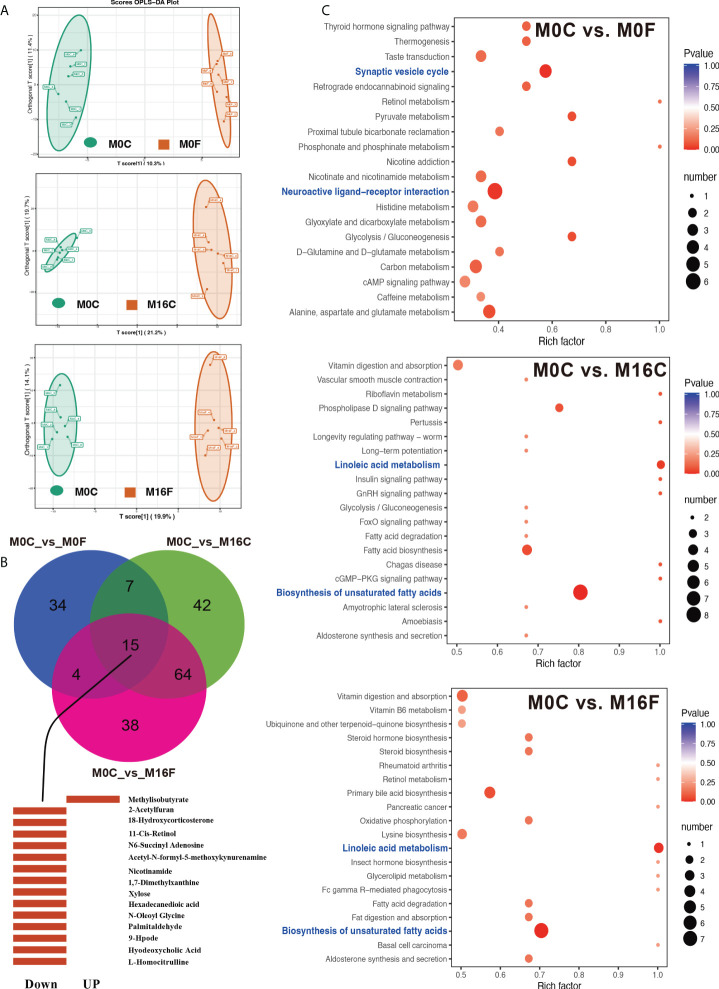
Folic acid changes the metabolome and pathways in the blood. OPLS-DA analyses of total blood metabolites among the comparisons of M0-OC vs. M0-OF, M0-OC vs. M16-OC, and M0-OC vs. M16-OF **(A)**. The mutual hub different metabolites among the three comparisons **(B)**. Top 20 enriched KEGG pathways based on the different metabolites **(C)**. Richness factor = the ratio of different metabolites to the number of total metabolites in each pathway. The pathways highlighted with blue are the significant enriched metabolic pathways (*P* < 0.05).

A total of 461 compounds were identified in the serum metabolome. The OPLS-DA score plots of the serum samples from the three comparisons are shown in **Figure 1A**. Compared to the M0C group, 60, 128, and 121 differentially expressed metabolites were identified in the liver of the M0F, M16C, and M16F groups (**Table S2**). In total, 15 differentially expressed mutual metabolites were found, of which 14 differentially expressed metabolites were downregulated and 1 were upregulated in the three FA groups compared to those in the M0C group (**Figure 1B**). The main downregulated differentially expressed metabolites were L-homocitrulline, hyodeoxycholic acid, 9-Hpode, palmitaldehyde, N-oleoyl glycine, hexadecanedioic acid, xylose, 1,7-dimethylxanthine, nicotinamide, N6-succinyl adenosine, 11-cis-retinol, acetyl-N-formyl-5-methoxykynurenamine, 18-hydroxycorticosterone, and 2-acetylfuran, and only methylisobutyrate was upregulated by FA feeding. The metabolome map revealed 2 enriched significant KEGG metabolic pathways in comparing M0C and M0F, including synaptic vesicle cycle and neuroactive ligand-receptor interaction (**Figure 1C**). For comparing M0C and M16C, M0C and M16F, two same significant KEGG metabolic pathways, including biosynthesis of unsaturated fatty acids and linoleic acid metabolism, were found.

### Liver and muscle transcriptome

**Figure 2:**
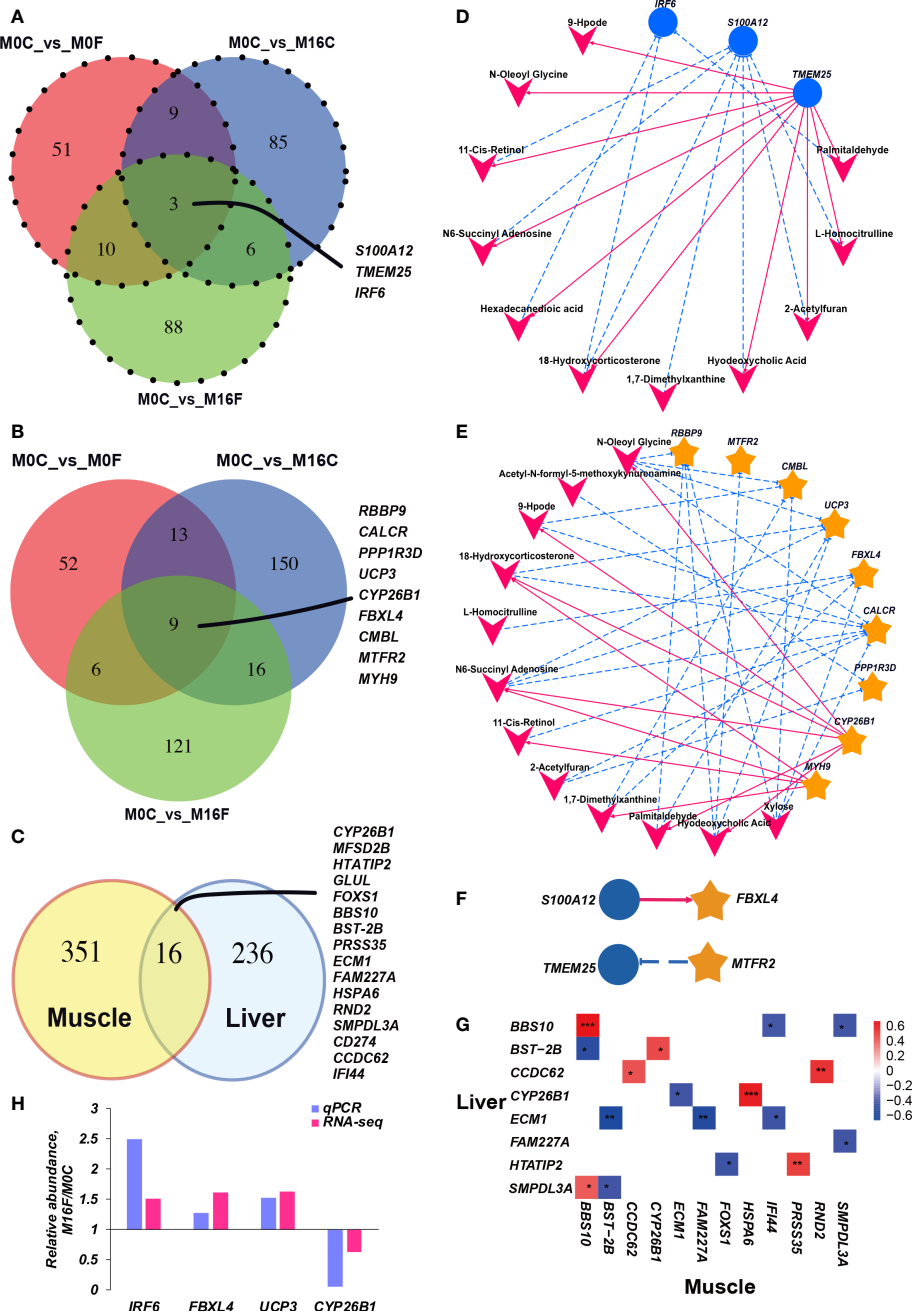
Cross-talk among the liver genes, muscle genes, and blood metabolites. Venn diagram illustrating the overlap of liver differential expressed genes between the three comparisons **(A)**. Venn diagram illustrating the overlap of muscle differential expressed genes between the three comparisons **(B)**. Venn diagram illustrating the overlap of differential expressed genes under the three comparisons between liver and muscle **(C)**. The association between hub differentially expressed genes in the muscle and blood hub different metabolites **(D)**. The association between hub differentially expressed genes in the liver and blood different metabolites **(E)**. The association between hub differentially expressed genes in the liver and muscle **(F)**. The correlation heatmap between mutual differentially expressed genes in the liver and muscle **(G)**. Each co-occurring pair between two items had an absolute Spearman tank correlation above 0.53 (red full-line, positive correlation; blue dotted line, negative correlation) with a *P*-value under 0.01. The blue circle indicates differential hepatic genes, the yellow star indicates differential muscle genes, and the V-shape indicates blood metabolites. The selected core genes (*IRF6* in the liver and *FBXL4*, *UCP3*, and *CYP26B1* in the muscle) verified by quantitative real-time PCR **(H)**.

RNA sequencing-based transcriptome analysis was performed to determine metabolic disorders in the liver. In total, 50.69 ± 3.69 and 51.78 ± 5.04 million clean sequence reads were obtained from the liver and muscle transcriptome, respectively. 31,565 and 30,637 genes were identified in the liver and muscle, respectively. The principal component analysis (PCA) score plots and volcano plots of the liver and muscle from the four groups were shown in **Figures S1**, **S2**, respectively. The PCA plots from both liver and muscle did not show a clear separation between the four groups. Compared to the M0C group, 238, 263, and 280 differentially expressed genes were identified in the liver of M0F, M16C, and M16F groups, and 179, 357, and 305 differentially expressed genes were identified in the muscle of M0F, M16C, and M16F groups. The mutual differentially expressed genes with annotated names in the liver among the three comparisons were *S100A12*, *TMEM25*, and *IRF6* (**Figure 2A**), and the mutual differentially expressed genes with annotated names in muscle among the three comparisons are *RBBP9, CYP26B1, CALCR, PPP1R3D, UCP3, FBXL4, CMBL, MTFR2, MYH9 *(**Figure 2B**). The mutual differentially expressed genes between liver and muscle were *CYP26B1, MFSD2B, HTATIP2, GLUL, FOXS1, BBS10, BST-2B, PRSS35, ECM1, FAM227A, HSPA6, RND2, SMPDL3A, CD274, CCDC62*, and* IFI44 *(**Figure 2C**).

### Correlation analysis

Inter-omics correlation networks of all the blood metabolome variables, liver and muscle transcriptomes that were mutually different among the three comparisons were established (**Figures 2D–G**). In the communication between liver and blood (**Figure 2D**), hepatic *IRF6* was negatively correlated with blood palmitaldehyde, 18-hydroxycorticosterone, hexadecanedioic acid. Hepatic *S100A12* was negatively correlated with blood L-homocitrulline, 2-acetylfuran, hyodeoxycholic acid, 1,7-dimethylxanthine, 18-hydroxycorticosterone, N6-succinyl adenosine, 11-cis-retinol. *TMEM25* was positively correlated with 2-acetylfuran, palmitaldehyde, L-homocitrulline, hexadecanedioic acid, N-oleoyl glycine, hyodeoxycholic acid, 18-hydroxycorticosterone, 9-Hpode, 11-cis-retinol, and N6-succinyl adenosine. In the connection between muscle and blood (**Figure 2E**), *CALCR* was negatively correlated with blood 2-acetylfuran, xylose, 11-cis-retinol, 9-Hpode, N-oleoyl glycine, 18-hydroxycorticosterone, and N6-succinyl adenosine. *CMBL* was negatively correlated with blood xylose, 1,7-dimethylxanthine, 9-Hpode, and N-oleoyl glycine. *CYP26B1 *was negatively correlated with blood N-oleoyl glycine, palmitaldehyde, N6-succinyl adenosine, hyodeoxycholic acid, 9-Hpode, and 18-hydroxycorticosterone. Only hepatic *S100A12* was positively correlated with muscle *FBXL4*, and hepatic *TMEM25* was negatively correlated with muscle *MTFR2* (**Figure 2F**). There are 16 mutual differentially expressed genes between liver and muscle. During these 16 genes, only *BBS10* and *CCDC62* have a strong positive correlation between liver and muscle (**Figure 2G**).

### qPCR validation for core genes

To validate the core genes based on RNA-seq results, mutual differentially expressed genes, including *IRF6* in the liver and *FBXL4*, *UCP3*, and *CYP26B1* in the muscle, were selected for further verification using qRT-PCR (**Figure 2H**). As expected, the abundances of *IRF6*, *FBXL4*, and *UCP3* were increased in the M16F group, and the expression level of *CYP26B1* was decreased in the M16F group compared to the M0C group, which was significantly different between these two groups and overall agreed with the RNA-seq results.

### Pathway analysis based on transcriptome

**Figure 3:**
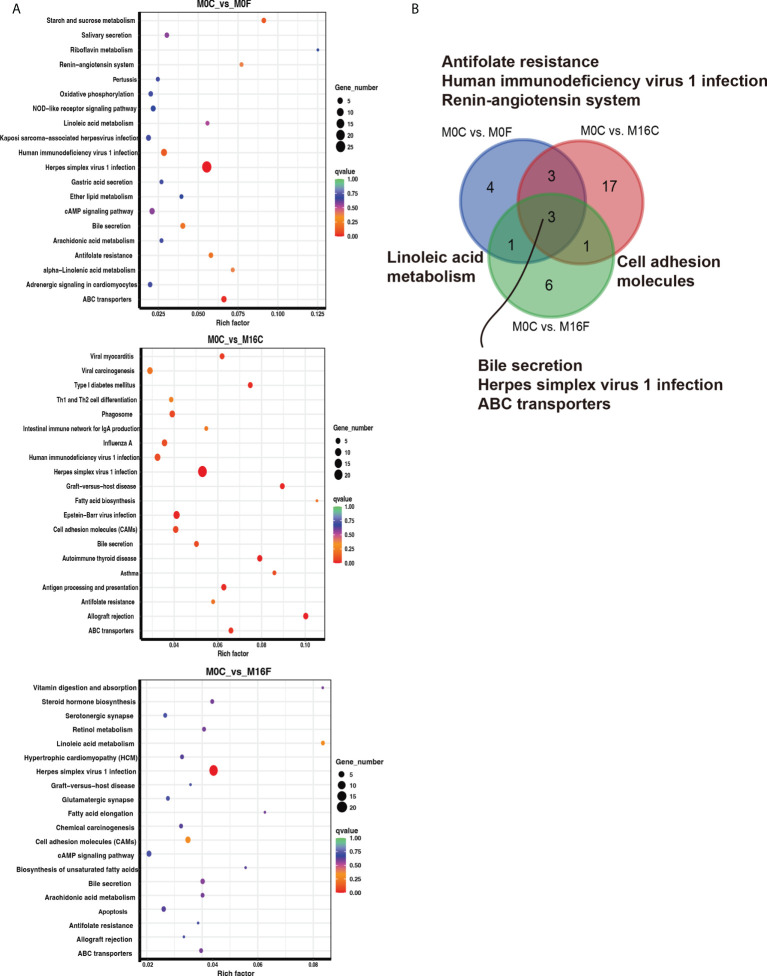
The hub affected KEGG pathways in the liver by folic acid supplementation. Top 20 enriched KEGG pathways based on the differentially expressed genes for the three comparisons in the liver **(A)**. Venn diagram illustrating the mutual liver pathways between the three comparisons **(B)**. Richness factor = the ratio of differentially expressed genes to the number of total genes in each pathway. The pathways highlighted with blue are the significant enriched metabolic pathways (*P* < 0.05).

**Figure 4:**
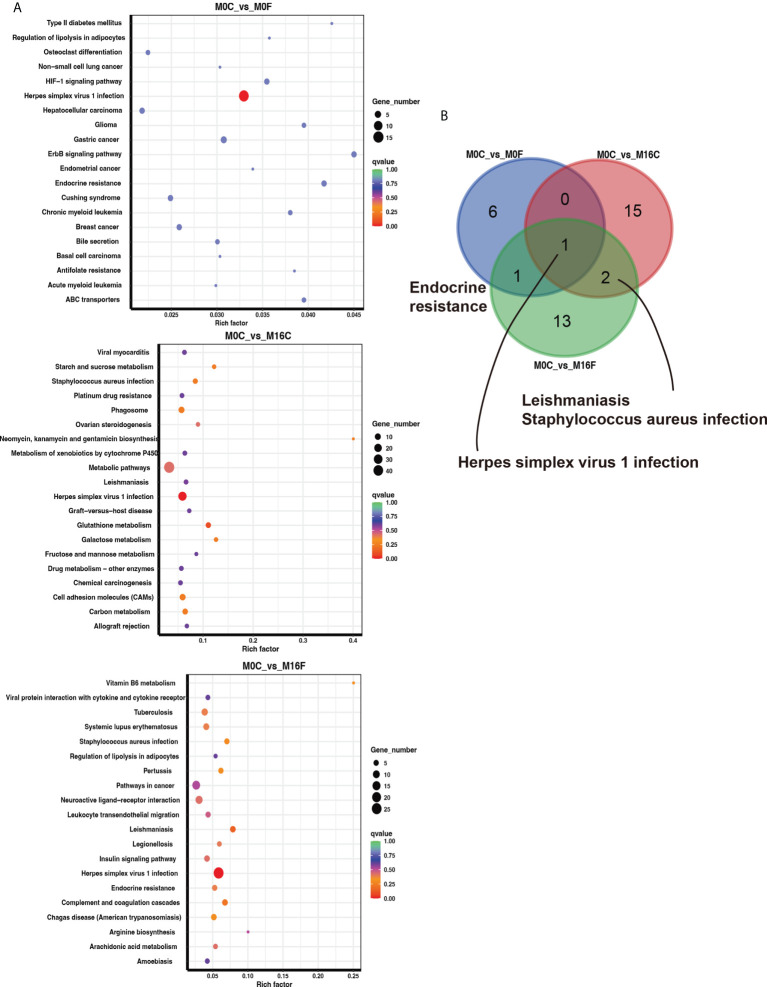
The hub affected KEGG pathways in the muscle by folic acid supplementation. Top 20 enriched KEGG pathways based on the differentially expressed genes for the three comparisons in the muscle **(A)**. Venn diagram illustrating the mutual muscle pathways between the three comparisons **(B)**. Richness factor = the ratio of differentially expressed genes to the number of total genes in each pathway. The pathways highlighted with blue are the significant enriched metabolic pathways (*P* < 0.05).

The enriched KEGG pathway analysis was performed to explore the biological function of the differentially expressed genes responding to the FA supplementation. 11, 24, and 11 functional different KEGG pathways were identified in the liver tissues from the comparisons of M0C vs. M0F, M0C vs. M16C, and M0C vs. M16F respectively (*P *< 0.05). The mutual different KEGG pathways among the four comparisons were bile secretion, herpes simplex virus 1 infection, and ABC transporters. Linoleic acid metabolism was a mutual pathway between M0C vs. M0F and M0C vs. M16F (**Figures 3A, B**). In the muscle, 8, 18, and 17 functional different KEGG pathways were identified from M0C vs. M0F, M0C vs. M16C, and M0C vs. M16F, respectively (*P* < 0.05). The mutual different KEGG pathway was herpes simplex virus 1 infection (**Figures 4A, B**).

## Discussion

**Figure 5:**
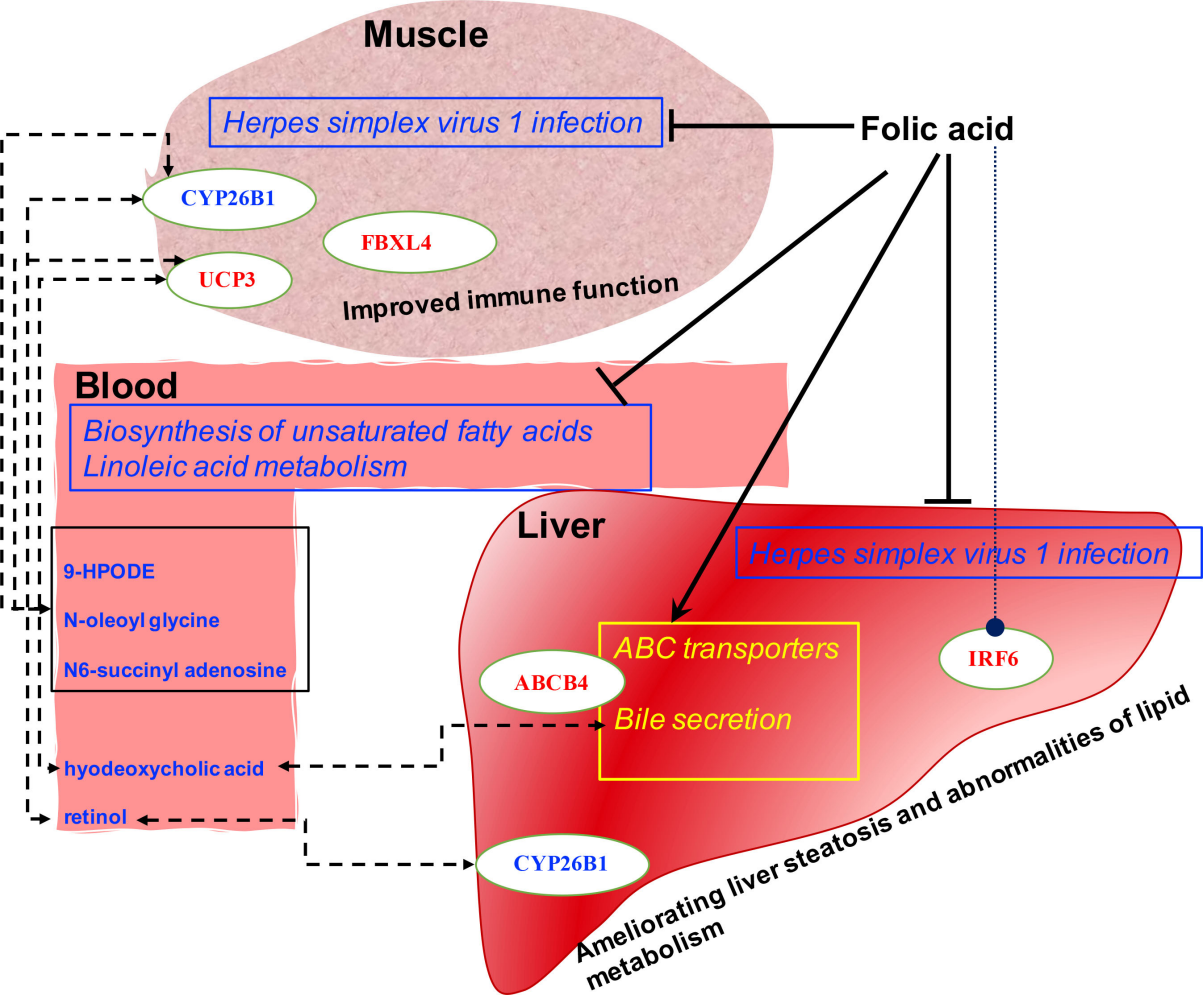
Proposed mechanism of folic acid’s function by the altered liver and muscle genes network and blood metabolic profiles. The upregulated and downregulated pathways are yellow and blue with squares, respectively. The upregulated and down-regulated hepatic and muscle genes and blood metabolites are labeled in red and blue, respectively. The dotted line indicates a potential biological communication.

The present study’s findings systematically revealed the FA’s effect on the liver-muscle axis using multi-omics, which provided clues regarding the potential protective roles of FA on the immune function of lamb. To the best of our knowledge, we identified potent crosstalk between the blood, liver, and muscle in ruminants for the first time. This crosstalk may be the potential mechanism of FA-induced host transcription and metabolic responses through maternal and offspring communication (**Figure 5**).

The liver transcriptome depended on maternal protein and FA intakes during pregnancy (21). The coated FA had stimulatory impacts on hepatic gene expression in Holstein bulls (14), indicating the roles of FA on hepatic gene transcription. The maternal supplementation with FA had defending effects on inflammation in Holstein calves (22). FA could protect host defense against the challenge of *Staphylococcus aureus* through activating cytoplasmic DNA sensing and tight junction pathway, indicating the FA’s potentially biological function in improving animal immune function (23). In the current study, the Herpes simplex virus 1 infection pathway was inhibited by feeding FA, which was enriched in both liver and muscle. Herpes simplex virus type 1 is a nuclear replicating enveloped virus, usually acquired through direct contact with infected lesions or body fluids (24). The recurrence rate of herpetic keratitis was lower in patients with a higher blood level of FA, indicating FA’s vital protective role in herpes simplex keratitis (25). The folate-conjugated herpes simplex virus type 1 G207 presents a folate receptor-targeted oncolytic virus with a potential therapeutic value and an increased anti-tumor efficiency (26). A recent study reported that the FA and its derivates like tetrahydrofolic acid and 5-methyl tetrahydrofolic acid against SARS-COV-2 (27). Therefore, FA might have an essential function in immunological enhancement in sheep based on the result of the current study.

Furthermore, we found three hepatic hub genes: upregulated *IRF6* and *S100A12* and downregulated *TMEM25*. Interferon regulatory factor 6, a molecular marker associated with the serum IL10 level, plays a functional role in regulating the immune system (28). The *IRF6* is significantly downregulated in high-fat diet-induced nonalcoholic fatty liver disease, a worldwide epidemic. IRF6 in hepatocytes is a protective factor in liver steatosis and abnormalities of lipid metabolism (29). In addition, an association of interaction between the *IRF6* gene and maternal FA intake with non-syndromic cleft lip/palate in Mexican Mestizos was found (30). The current study found that the upregulated IRF6 by feeding FA confirms the FA’s beneficial role in hepatic immune function and metabolism and indicates that the IRF6 might be the targeted gene of FA in hepatocytes. S100 calcium binding protein A12 is a pro-inflammatory factor associated with non-infectious inflammatory diseases (31). *S100A12* gene silencing decreased serum levels of anti-inflammatory/pro-inflammatory cytokines in septic rats (32). However, the down-regulation of *TMEM25* is colorectal cancer-related (33), which needs further studies to clarify the molecular mechanism of *TMEM25* in sheep.

*CYP26B1*, a mutual gene between liver and muscle transcriptome, was down-regulated by feeding FA. Similarly, *CYP26B1* was down-regulated by dietary vitamin E supplementation in sheep (34), similar to the finding of this study. Retinoic acid and CYP26B1 are critical regulators of osteogenesis in the axial skeleton (35). Retinoic acid is synthesized intracellularly from its precursor, vitamin A (or retinol) (36). CYP26B1 can regulate retinoic acid-dependent signals in T cells and can be inhibited by transforming growth factor-β (TGF-β) (37). Thus, the down-regulated levels of *CYP26B1* mRNA abundance in muscle and blood 11-cis-retinol are consistent with the enhanced immune function of feeding FA. MYH9 encodes the heavy chain of non-muscle myosin IIA, a cytoplasmic myosin that plays a significant role in human development and disease (38, 39). RBBP9 can suppress the TGF-β signaling required for a concomitant decrease in the integrity of tumor cell-cell junctions, indicating its protective role for body health (40). The PPP1R3D can regulate the structure of glycogen, and the deficiency of PPP1R3D preferentially inhibits neuronal and cardiac Lafora body formation in a mouse model of the fatal epilepsy Lafora disease (41). FBXL4 has a crucial function in development, metabolism, and mitochondrial dynamics and may be used to develop novel therapies for mitochondrial encephalopathy (42). Thus, the decreased abundances of *CYP26B1* and *MYH9* and increased abundances of *RBBP9*, *PPP1R3D*, and *FBXL4* in muscle, confirmed the improved immune function of sheep by supplementation of FA.

On the other hand, FA increased the bile flow, bile acid synthesis from cholesterol, and bile acid excretion *via* feces (43). It was suggested that FA is an essential therapeutic intervention in dyslipidemia caused by cholestasis, protecting the liver against cholestasis (44). The enhanced bile synthesis pathway in the liver from the current study confirms the function of FA on bile acid metabolism in sheep. In addition, the blood hyodeoxycholic acid, a primary secondary bile acid, was reduced by feeding FA from the maternal or offspring diet. A significantly negative correlation between blood hyodeoxycholic acid and muscle uncoupling protein 3 (UCP3) gene was observed. Hyodeoxycholic acid can solubilize dietary fat and promote fat absorption and glucose homeostasis (45). *UCP3* gene was an important candidate gene for regulating intramuscular fat content in chicken breast muscle (46). We did find the changed lipid metabolism by decreasing serum cholesterol parameters and increasing fat deposition in tail or omentum from our previous studies (5, 6). Two enriched metabolic pathways, biosynthesis of unsaturated fatty acids and linoleic acid metabolism, were down-regulated by feeding FA from the maternal diet solely and maternal and offspring diet together, indicating the crucial roles of FA on lipid metabolism. However, no information indicated the relationship between UCP3 and fat metabolism in sheep. Thus, we estimated that the FA has potential roles in alleviating fat and unsaturated fatty acids deposition in sheep by regulating the association between hyodeoxycholic acid and UCP3.

9-Hpode, N-oleoyl glycine, and N6-succinyl adenosine were positively correlated with *CYP26B1* but negatively correlated with *UCP3*. Blood 9-Hpode is the peroxidation product of linoleic acid (47). 9-Hpode is the main product of lipid peroxidation, and the increased 9-Hpode can cause some inflammatory reactions, such as atherosclerosis (48) and rheumatoid arthritis (49). The decreased blood 9-Hpode by adding FA to the maternal or offspring diet might indicate the reduced peroxidation of linoleic acid and prevent these diseases, mainly because that FA can scavenge free radicals in the body to prevent oxidative damage (50, 51). In addition, N-oleoyl glycine and N6-succinyl adenosine were down-regulated by feeding FA. The N-oleoyl glycine can potentially increase insulin sensitivity and suppress obesity and diabetes (52). The blood concentration of N6-succinyl adenosine was a feasible predictor of chronic renal failure in rat plasma (53). Homocitrulline is a biomarker for differentiating acute from chronic renal failure (54). The increased serum homocitrulline concentration is associated with the severity of coronary artery disease (55). Thus, these reduced blood hub differential metabolites by feeding FA from either maternal or offspring diets also prove their protection roles in improved immune function in sheep.

In conclusion, a multi-omics assessment of the blood, liver, and muscle systematically revealed the molecular biological mechanisms of FA on lamb health and lipid metabolic performance. The inhibited pathway of herpes simplex virus 1 infection might play a crucial role of FA in enhancing lamb health. Moreover, the enhanced bile secretion pathway in the liver by FA from maternal or offspring diet and the inhibited blood biosynthesis of unsaturated fatty acids and linoleic acid metabolism by FA from offspring diet might contribute to the changed fat deposition and lipid metabolism in sheep. We identified the possible hub genetic biomarkers *IRF6* in the liver and *CYP26B1*, *FBXL4*, and *UCP3* in the muscle, and potential folic related functional biological or metabolic intermediates including blood hyodeoxycholic acid, 9-Hpode, N-oleoyl glycine, and N6-succinyl adenosine, as being responsible for FA’s function in lamb. Our results provide a systemic picture of FA’s roles in immunological enhancement in a lamb model, which enhances the understanding of FA from either maternal and offspring diet in improving animal health in the future.

## Data availability statement

The datasets presented in this study can be found in online repositories. The names of the repository/repositories and accession number(s) can be found below: https://www.ncbi.nlm.nih.gov/, PRJNA822866 https://www.ncbi.nlm.nih.gov/, PRJNA822707.

## Ethics statement

The animal study was reviewed and approved by Animal Care and Use Committee of China Agricultural University.

## Author contributions

BW and HLL conceived and designed the study; BW wrote the manuscript; BW, HQL, ZL, and BoW carried out the animal experiment, laboratory work, data analysis and bioinformatic analyses; BoW and ZL did the partial data analysis; HZ did the qPCR test; BZ did the Figures visualization; BW and HLL revised the manuscript. All the authors gave final approval for the publication of the manuscript and agree to be held accountable for the work performed therein.

## Acknowledgments

We greatly acknowledge the grants supported by the earmarked fund for CARS-38 and the National Key Research and Development Program of China (No. 2018YFD0500402).

## Conflict of interest

The authors declare that the research was conducted in the absence of any commercial or financial relationships that could be construed as a potential conflict of interest.

## Publisher’s note

All claims expressed in this article are solely those of the authors and do not necessarily represent those of their affiliated organizations, or those of the publisher, the editors and the reviewers. Any product that may be evaluated in this article, or claim that may be made by its manufacturer, is not guaranteed or endorsed by the publisher.
