# Supplementary material for: Integrative network analysis revealed the molecular function of folic acid on immunological enhancement in a sheep model
Source: Front Immunol. 2022 Aug 12;13:913854. doi: 10.3389/fimmu.2022.913854 (PMC9412826; doi:10.3389/fimmu.2022.913854)
Supplement: Supplementary file 1 [file DataSheet_1.docx]

***Supplementary Material***

**Integrative network analysis revealed the molecular function of folic acid on immunological enhancement in a sheep model**

*Bing Wang, Heqiong Li, Zhen Li, Bo Wang, Han Zhang, Boyan Zhang, and Hailing Luo**

State Key Laboratory of Animal Nutrition, College of Animal Science and Technology, China Agricultural University, Beijing 100193, P. R. China

Running title: Immunological mechanism of folic acid using multi-omics

*Correspondence: [luohailing@cau.edu.cn](mailto:luohailing@cau.edu.cn)

**Table S1**

Gene primers used for quantitative real time-PCR.

| Gene | Forward primer (5'-3') | Reverse primer (5'-3') |
| --- | --- | --- |
| *ACTB* | CATCGTCCACCGCAAAT | GCCATGCCAATCTCATCTC |
| *IRF6* | CCCAGGCACCTATACAGC | TGTCCGTACTCCTTCCCA |
| *FBXL4* | AAATAGCAGAACTCACGATGG | TGGAGCAGAAAACAAAAGGT |
| *UCP3* | CCCCTTCGACTGTATGCTG | CCCGTTTCATCTGCTCGT |
| *CYP26B1* | CCCCAAAGGCTGGAGTGT | CGCCGAAAGGGAGGTAAT |

|  |
| --- |
|  |

**Table S2**

Identification of different metabolites between each two groups in the blood^1^.

| Compounds | Relative concentration | | VIP | Log_2_FC |
| --- | --- | --- | --- | --- |
| M0C vs. M0F | M0C | M0F |  |  |
| Glycochenodeoxycholic acid | 9.04E+05 | 3.60E+06 | 1.45 | 2.00 |
| Vanillin | 3.22E+04 | 7.84E+04 | 1.02 | 1.28 |
| 3-Indolepropionic acid | 1.92E+05 | 3.11E+05 | 1.78 | 0.69 |
| N-Formylmethionine | 1.95E+05 | 3.12E+05 | 1.53 | 0.68 |
| Serotonin | 5.49E+06 | 8.68E+06 | 1.44 | 0.66 |
| 2-Hydroxyisocaproic acid | 7.88E+03 | 1.23E+04 | 2.36 | 0.64 |
| Deoxyguanosine | 4.67E+04 | 6.87E+04 | 1.09 | 0.56 |
| Oxaloacetic acid | 6.59E+04 | 9.63E+04 | 1.97 | 0.55 |
| Lumichrome | 3.74E+04 | 5.30E+04 | 2.46 | 0.50 |
| 4-Hydroxy-L-Glutamic acid | 7.20E+04 | 1.00E+05 | 1.60 | 0.47 |
| Glycine | 1.67E+05 | 2.27E+05 | 1.84 | 0.44 |
| L-Glutamic acid | 1.88E+06 | 2.48E+06 | 1.14 | 0.40 |
| Sebacate | 8.81E+04 | 1.16E+05 | 1.18 | 0.39 |
| 3-Methylsalicylic acid | 2.43E+04 | 3.18E+04 | 1.30 | 0.38 |
| N-Methyl-L-Glutamate | 3.59E+05 | 4.69E+05 | 1.61 | 0.38 |
| N-Acetylphenylalanine | 5.08E+04 | 6.62E+04 | 1.03 | 0.38 |
| 3,4,5-Trimethoxycinnamic acid | 2.41E+04 | 3.13E+04 | 1.02 | 0.38 |
| 5-Oxoproline | 6.45E+05 | 8.37E+05 | 1.29 | 0.38 |
| Diphenyl ether | 8.93E+04 | 1.15E+05 | 2.42 | 0.37 |
| Methylisobutyrate | 2.95E+05 | 3.79E+05 | 1.50 | 0.36 |
| N-Acetyl-L-Leucine | 6.69E+04 | 8.55E+04 | 1.30 | 0.35 |
| N-Glycyl-L-Leucine | 1.54E+05 | 1.95E+05 | 1.34 | 0.34 |
| Diethanolamine | 5.70E+04 | 7.20E+04 | 1.13 | 0.34 |
| Trans-4-Hydroxy-L-Proline | 4.72E+04 | 5.94E+04 | 1.73 | 0.33 |
| D-Fructose 6-Phosphate-Disodium Salt | 5.15E+04 | 6.36E+04 | 1.08 | 0.30 |
| Pyrrolidine | 9.48E+05 | 1.17E+06 | 1.20 | 0.30 |
| Nα-Acetyl-L-glutamine | 2.75E+05 | 3.37E+05 | 1.44 | 0.29 |
| 2'-Deoxycytidine 5'-Diphosphate | 1.66E+06 | 2.02E+06 | 1.06 | 0.29 |
| L-Aspartic acid | 1.59E+05 | 1.93E+05 | 1.45 | 0.28 |
| γ-Aminobutyric acid | 3.24E+04 | 3.90E+04 | 1.20 | 0.27 |
| Betaine | 8.24E+07 | 9.92E+07 | 1.68 | 0.27 |
| 18-Hydroxycorticosterone | 1.55E+06 | 1.28E+06 | 2.55 | -0.27 |
| L-Homocitrulline | 3.42E+05 | 2.83E+05 | 1.16 | -0.28 |
| Syringic acid | 7.32E+05 | 6.04E+05 | 1.39 | -0.28 |
| 9-Hpode | 3.39E+04 | 2.78E+04 | 1.62 | -0.29 |
| 2-Pyrrolidinone | 6.87E+05 | 5.60E+05 | 1.11 | -0.29 |
| N6-Succinyl adenosine | 8.08E+05 | 6.55E+05 | 1.84 | -0.30 |
| Nicotinamide | 2.09E+07 | 1.69E+07 | 1.83 | -0.30 |
| N-Oleoyl glycine | 8.15E+03 | 6.57E+03 | 1.93 | -0.31 |
| 3,3',5'-Triiodothyronine | 2.02E+03 | 1.61E+03 | 1.10 | -0.33 |
| 3,3',5-Triiodo-L-Thyronine | 4.62E+04 | 3.66E+04 | 2.12 | -0.33 |
| Hexadecanedioic acid | 1.55E+06 | 1.23E+06 | 1.80 | -0.33 |
| Palmitaldehyde | 7.30E+07 | 5.75E+07 | 2.65 | -0.34 |
| 2-Furoylglycine | 1.88E+05 | 1.47E+05 | 1.06 | -0.35 |
| 2-Acetylfuran | 3.01E+07 | 2.35E+07 | 2.44 | -0.36 |
| Hyodeoxycholic acid | 1.04E+05 | 8.13E+04 | 1.80 | -0.36 |
| Menaquinone | 5.35E+04 | 4.16E+04 | 1.69 | -0.36 |
| 11-Cis-Retinol | 6.14E+04 | 4.78E+04 | 2.08 | -0.36 |
| 1,7-Dimethylxanthine | 2.47E+04 | 1.90E+04 | 1.92 | -0.38 |
| Xylose | 3.40E+04 | 2.59E+04 | 1.44 | -0.39 |
| L-Lactic acid | 8.41E+05 | 6.41E+05 | 2.12 | -0.39 |
| 2,6-Dihydroxypurine | 1.08E+06 | 8.16E+05 | 1.44 | -0.40 |
| DHA [4Z,7Z,10Z,13Z,16Z,19Z-docosahexaenoic acid] | 8.09E+03 | 6.04E+03 | 1.52 | -0.42 |
| N-Acetylhistamine | 1.07E+05 | 7.93E+04 | 1.36 | -0.43 |
| Acetyl-N-formyl-5-methoxykynurenamine | 1.91E+04 | 1.41E+04 | 2.08 | -0.44 |
| Guanidinoethyl sulfonate | 3.76E+04 | 2.71E+04 | 1.58 | -0.47 |
| DL-Carnitine | 8.68E+06 | 6.06E+06 | 2.12 | -0.52 |
| L-Carnitine | 2.78E+06 | 1.93E+06 | 1.49 | -0.53 |
| Phenylacetic acid | 1.77E+03 | 1.20E+03 | 1.97 | -0.56 |
| N'-Formylkynurenine | 7.29E+05 | 4.51E+05 | 1.16 | -0.69 |
|  |  |  |  |  |
| M0C vs. M0F | M0C | M16C | VIP | Log2FC |
| Adenosine | 7.08E+06 | 1.79E+07 | 1.42 | 1.33 |
| Uridine 5-Monophosphate | 5.30E+04 | 8.58E+04 | 1.73 | 0.69 |
| 4-Hydroxy-L-Glutamic acid | 7.20E+04 | 1.15E+05 | 1.12 | 0.67 |
| L-Glutamic acid | 1.88E+06 | 2.82E+06 | 1.16 | 0.59 |
| Phenylpyruvate | 4.59E+04 | 6.58E+04 | 1.50 | 0.52 |
| Methylisobutyrate | 2.95E+05 | 3.98E+05 | 1.27 | 0.43 |
| Barbituric acid | 8.00E+06 | 1.03E+07 | 1.61 | 0.36 |
| Riboflavin | 2.57E+05 | 3.29E+05 | 1.14 | 0.35 |
| Thiamine triphosphate | 7.12E+04 | 9.08E+04 | 2.09 | 0.35 |
| (E)-2-Octen-1-ol | 6.95E+06 | 8.43E+06 | 1.35 | 0.28 |
| Terephthalic acid | 1.57E+06 | 1.31E+06 | 1.63 | -0.27 |
| Lysopc 15:0 | 2.26E+07 | 1.87E+07 | 1.01 | -0.27 |
| Creatine | 8.48E+05 | 6.99E+05 | 1.26 | -0.28 |
| alpha-Terpinene | 2.33E+05 | 1.92E+05 | 2.00 | -0.28 |
| Lysopc 18:3 | 7.75E+07 | 6.36E+07 | 1.14 | -0.28 |
| Adenine | 4.13E+06 | 3.39E+06 | 1.14 | -0.28 |
| Punicic acid | 1.90E+07 | 1.56E+07 | 2.14 | -0.28 |
| EPA [5Z,8Z,11Z,14Z,17Z-eicosapentaenoic acid] | 1.21E+05 | 9.93E+04 | 1.24 | -0.29 |
| Hexadecanoic acid (C16:0) | 3.16E+06 | 2.59E+06 | 1.43 | -0.29 |
| Β-Nicotinamide mononucleotide | 2.77E+04 | 2.26E+04 | 1.63 | -0.29 |
| 2-(3,4-dimethoxyphenyl)ethanamine | 6.87E+07 | 5.60E+07 | 1.21 | -0.29 |
| 1-Naphthol | 4.26E+04 | 3.46E+04 | 1.82 | -0.30 |
| L-Arginine | 4.65E+06 | 3.77E+06 | 1.61 | -0.30 |
| N-acetylornithine | 4.31E+06 | 3.49E+06 | 1.63 | -0.30 |
| Butylparaben | 3.80E+03 | 3.05E+03 | 1.24 | -0.32 |
| L-Carnosine | 2.43E+06 | 1.95E+06 | 1.08 | -0.32 |
| 2'-Deoxyuridine | 8.23E+04 | 6.61E+04 | 1.02 | -0.32 |
| 2,4-Dihydroxypteridine | 2.65E+05 | 2.12E+05 | 1.18 | -0.32 |
| Gamma-Glu-Leu | 1.49E+05 | 1.19E+05 | 1.17 | -0.33 |
| Homovanillic acid | 2.13E+07 | 1.68E+07 | 1.26 | -0.34 |
| 2-Hydroxycinnamic acid | 6.25E+07 | 4.94E+07 | 1.26 | -0.34 |
| Methylcysteine | 2.37E+07 | 1.87E+07 | 1.28 | -0.34 |
| Isonicotinic acid | 6.67E+06 | 5.26E+06 | 1.17 | -0.34 |
| beta-Cubebene | 4.99E+04 | 3.93E+04 | 1.92 | -0.34 |
| Lysope 18:1 | 4.27E+06 | 3.36E+06 | 1.34 | -0.34 |
| 2,4-Di-tert-butylphenol | 4.52E+04 | 3.53E+04 | 1.91 | -0.35 |
| 4-tert-Octylphenol | 6.76E+05 | 5.28E+05 | 1.98 | -0.36 |
| L-Theanine | 3.94E+06 | 3.08E+06 | 1.06 | -0.36 |
| Nicotinic acid | 9.25E+05 | 7.22E+05 | 1.28 | -0.36 |
| Syringic acid | 7.32E+05 | 5.71E+05 | 1.15 | -0.36 |
| 1-Methylguanine | 2.01E+04 | 1.57E+04 | 1.05 | -0.36 |
| Nicotinamide | 2.09E+07 | 1.62E+07 | 1.19 | -0.36 |
| Allantoin | 2.74E+06 | 2.13E+06 | 1.14 | -0.36 |
| 2,5-Dihydroxy Benzoic Acid | 8.68E+04 | 6.69E+04 | 1.92 | -0.37 |
| 3-Hydroxyhippuric Acid | 5.00E+05 | 3.84E+05 | 1.16 | -0.38 |
| Pantothenate | 8.61E+05 | 6.60E+05 | 1.68 | -0.38 |
| L-Citrulline | 1.51E+07 | 1.16E+07 | 1.22 | -0.39 |
| P-Coumaric acid | 1.10E+07 | 8.41E+06 | 1.29 | -0.39 |
| 5,6-Dihydro-5-Methyluracil | 3.99E+05 | 3.05E+05 | 1.01 | -0.39 |
| Oxaloacetic acid | 6.59E+04 | 5.01E+04 | 1.48 | -0.39 |
| 2-Acetylfuran | 3.01E+07 | 2.29E+07 | 1.56 | -0.39 |
| L-Histidine | 8.17E+05 | 6.20E+05 | 1.49 | -0.40 |
| 4-Guanidinobutyric acid | 2.42E+06 | 1.83E+06 | 1.18 | -0.40 |
| Lysope 16:0 | 2.11E+06 | 1.60E+06 | 1.61 | -0.40 |
| Lumichrome | 3.74E+04 | 2.82E+04 | 1.49 | -0.40 |
| Lysopc 17:0 | 1.58E+07 | 1.19E+07 | 1.20 | -0.41 |
| D-Arabinose | 3.14E+04 | 2.36E+04 | 1.19 | -0.41 |
| Thiamine | 1.22E+05 | 9.13E+04 | 1.16 | -0.42 |
| Acetyl-N-formyl-5-methoxykynurenamine | 1.91E+04 | 1.43E+04 | 1.20 | -0.42 |
| 2-Aminoadipic acid | 5.86E+05 | 4.37E+05 | 1.03 | -0.42 |
| L-Thyroxine | 2.25E+06 | 1.68E+06 | 1.24 | -0.43 |
| Pyrrole-2-Carboxylic acid | 1.37E+04 | 1.02E+04 | 1.14 | -0.43 |
| 4-Hydroxy-2-Oxoglutaric acid | 5.91E+04 | 4.38E+04 | 1.05 | -0.43 |
| Palmitaldehyde | 7.30E+07 | 5.40E+07 | 1.79 | -0.43 |
| N-Acetyl-D-Glucosamine | 4.40E+04 | 3.24E+04 | 1.19 | -0.44 |
| L-Cystine | 5.02E+04 | 3.70E+04 | 1.52 | -0.44 |
| DL-o-Tyrosine | 4.72E+06 | 3.47E+06 | 1.31 | -0.45 |
| Lysopc 18:0 | 1.66E+08 | 1.21E+08 | 1.55 | -0.45 |
| P–Hydroxyphenyl acetic acid | 2.33E+05 | 1.69E+05 | 1.07 | -0.46 |
| Lysope 18:0 | 2.98E+05 | 2.15E+05 | 1.67 | -0.47 |
| Lysopg 18:1 | 6.39E+04 | 4.61E+04 | 1.90 | -0.47 |
| D-Glucose | 7.77E+04 | 5.61E+04 | 1.14 | -0.47 |
| Lysopa 16:0 | 4.69E+05 | 3.37E+05 | 1.79 | -0.48 |
| PAF C-16 | 1.86E+08 | 1.34E+08 | 1.62 | -0.48 |
| 2,6-Diaminooimelic acid | 6.57E+04 | 4.67E+04 | 1.43 | -0.49 |
| N-Acetylthreonine | 3.44E+04 | 2.44E+04 | 1.60 | -0.50 |
| N-(2-Methylbenzoyl)glycine | 7.32E+05 | 5.17E+05 | 1.38 | -0.50 |
| Arachidic Acid(C20:0) | 2.49E+06 | 1.75E+06 | 1.62 | -0.51 |
| Lysops 22:6 | 1.35E+04 | 9.52E+03 | 1.78 | -0.51 |
| 4-Hydroxyhippurate | 5.11E+04 | 3.59E+04 | 1.79 | -0.51 |
| Mandelic Acid | 4.72E+05 | 3.30E+05 | 1.10 | -0.52 |
| 1,7-Dimethylxanthine | 2.47E+04 | 1.72E+04 | 1.34 | -0.52 |
| Ubiquinone-1 | 4.19E+04 | 2.92E+04 | 1.12 | -0.52 |
| N-Acetyl-L-Tyrosine | 1.85E+04 | 1.28E+04 | 1.68 | -0.53 |
| D-Glucoronic acid | 7.04E+04 | 4.88E+04 | 1.41 | -0.53 |
| 5-Methoxytryptophol | 1.28E+06 | 8.72E+05 | 1.08 | -0.56 |
| Dodecanedioic acid | 2.10E+05 | 1.42E+05 | 1.08 | -0.56 |
| Kinurenine | 1.42E+07 | 9.50E+06 | 1.03 | -0.58 |
| Salicyluric acid | 4.82E+04 | 3.21E+04 | 1.82 | -0.58 |
| O-Phospho-L-Serine | 3.25E+04 | 2.14E+04 | 1.21 | -0.60 |
| D-Homocysteine | 3.73E+06 | 2.45E+06 | 1.06 | -0.60 |
| Cyclic Amp | 3.48E+05 | 2.26E+05 | 1.03 | -0.62 |
| 4-Hydroxybenzyl alcohol | 4.45E+04 | 2.88E+04 | 1.72 | -0.63 |
| Hexadecanedioic acid | 1.55E+06 | 1.01E+06 | 1.69 | -0.63 |
| Taurochenodesoxycholic acid | 1.20E+05 | 7.73E+04 | 2.03 | -0.63 |
| 6β-hydroxytestosterone | 3.36E+03 | 2.14E+03 | 1.65 | -0.65 |
| L-Cystathionine | 7.20E+04 | 4.55E+04 | 1.26 | -0.66 |
| 18-Hydroxycorticosterone | 1.55E+06 | 9.77E+05 | 2.13 | -0.67 |
| Hyodeoxycholic acid | 1.04E+05 | 6.52E+04 | 1.79 | -0.68 |
| tryptophan betaine | 6.75E+06 | 4.19E+06 | 1.06 | -0.69 |
| Elaidic Acid (C18:1N9T) | 4.80E+03 | 2.98E+03 | 1.44 | -0.69 |
| Lysopa 18:0 | 7.23E+04 | 4.43E+04 | 1.89 | -0.71 |
| N6-Succinyl adenosine | 8.08E+05 | 4.83E+05 | 1.77 | -0.74 |
| 9-Hpode | 3.39E+04 | 2.01E+04 | 1.65 | -0.75 |
| Xylose | 3.40E+04 | 2.01E+04 | 1.64 | -0.75 |
| Ureidoisobutyric acid | 4.39E+04 | 2.56E+04 | 1.29 | -0.78 |
| L-Homocitrulline | 3.42E+05 | 1.99E+05 | 1.77 | -0.79 |
| INDOLE-3-CARBINOL | 2.19E+05 | 1.24E+05 | 1.13 | -0.82 |
| Isoquinoline | 2.25E+05 | 1.27E+05 | 1.11 | -0.83 |
| Vanillylmandelic acid | 1.88E+04 | 1.06E+04 | 1.88 | -0.83 |
| Indole-3-Carboxaldehyde | 1.32E+05 | 7.39E+04 | 1.11 | -0.83 |
| 11-Cis-Retinol | 6.14E+04 | 3.44E+04 | 1.88 | -0.84 |
| Linoleic Acid (C18:2N6C) | 2.15E+06 | 1.17E+06 | 1.95 | -0.88 |
| 2,6-Dihydroxypurine | 1.08E+06 | 5.76E+05 | 1.61 | -0.90 |
| 12-Hete | 4.93E+03 | 2.57E+03 | 1.34 | -0.94 |
| N'-Formylkynurenine | 7.29E+05 | 3.79E+05 | 1.01 | -0.95 |
| 3-Ureidopropionate | 5.34E+04 | 2.77E+04 | 1.60 | -0.95 |
| (±)12-HETE [(±)12-hydroxy-5Z,8Z,10E,14Z-eicosatetraenoic acid] | 1.25E+04 | 6.46E+03 | 1.45 | -0.96 |
| Α-Linolenic acid(C18:3N3) | 1.61E+04 | 7.97E+03 | 1.78 | -1.01 |
| Dodecanoic acid(C12:0) | 2.70E+05 | 1.32E+05 | 2.09 | -1.03 |
| γ-Linolenic acid(C18:3N6) | 1.34E+04 | 6.33E+03 | 1.92 | -1.08 |
| Uridine 5'-Diphospho-N-Acetylgalactosamine | 1.92E+04 | 8.77E+03 | 1.98 | -1.13 |
| Palmitoleic acid (C16:1) | 1.06E+06 | 4.67E+05 | 2.08 | -1.18 |
| 13-HOTrE [13S-hydroxy-9Z,11E,15Z-octadecatrienoic acid] | 4.02E+04 | 1.74E+04 | 2.02 | -1.21 |
| N-Oleoyl glycine | 8.15E+03 | 3.17E+03 | 1.86 | -1.36 |
| Cis-11,14,17-Eicosatrienoic Acid (C20:3) | 1.50E+05 | 5.72E+04 | 2.05 | -1.39 |
| P-Aminohippuric acid | 2.95E+05 | 1.08E+05 | 1.27 | -1.45 |
| 8,15-Dihete | 7.53E+04 | 2.24E+04 | 2.04 | -1.75 |
|  |  |  |  |  |
| M0C vs. M16F | M0C | M16F | VIP | Log2FC |
| 2-(Methylthio)ethanol | 2.67E+05 | 4.64E+05 | 1.606127 | 0.798409 |
| Raffinose | 8.85E+03 | 1.26E+04 | 1.318496 | 0.515064 |
| Methylisobutyrate | 2.95E+05 | 4.04E+05 | 1.036699 | 0.452898 |
| Barbituric acid | 8.00E+06 | 1.08E+07 | 1.504736 | 0.428427 |
| (E)-2-Octen-1-ol | 6.95E+06 | 9.21E+06 | 1.389494 | 0.407017 |
| Ethylsalicylate | 3.58E+05 | 4.65E+05 | 1.816712 | 0.374591 |
| Porphobilinogen | 3.47E+05 | 4.49E+05 | 1.542814 | 0.372764 |
| N-Nitrosodiethylamine | 4.57E+05 | 5.89E+05 | 1.045379 | 0.364583 |
| Riboflavin | 2.57E+05 | 3.28E+05 | 1.019534 | 0.350455 |
| 6-Methyl-5-hepten-2-one | 1.20E+06 | 1.49E+06 | 1.326591 | 0.30692 |
| 3-Methylsalicylic acid | 2.43E+04 | 2.99E+04 | 1.616871 | 0.296712 |
| N-methylalanine | 1.21E+06 | 1.48E+06 | 1.502352 | 0.293205 |
| Phenylpyruvate | 4.59E+04 | 5.60E+04 | 1.025544 | 0.288053 |
| Methylmalonic acid | 1.51E+06 | 1.81E+06 | 1.065023 | 0.265351 |
| Nicotinamide | 2.09E+07 | 1.74E+07 | 1.192598 | -0.26459 |
| 2,4-Di-tert-butylphenol | 4.52E+04 | 3.75E+04 | 2.09572 | -0.2676 |
| Indole-3-acetamide | 6.05E+05 | 5.01E+05 | 1.010678 | -0.27175 |
| Nicotinic acid | 9.25E+05 | 7.65E+05 | 1.058279 | -0.27321 |
| 4-Hydroxyhippurate | 5.11E+04 | 4.19E+04 | 1.293013 | -0.28591 |
| Terephthalic acid | 1.57E+06 | 1.29E+06 | 1.397241 | -0.2866 |
| Xanthosine | 3.99E+05 | 3.27E+05 | 1.257765 | -0.28723 |
| DHA [4Z,7Z,10Z,13Z,16Z,19Z-docosahexaenoic acid] | 8.09E+03 | 6.63E+03 | 1.224837 | -0.28738 |
| P-Coumaric acid | 1.10E+07 | 9.02E+06 | 1.028772 | -0.28742 |
| N-Acetylthreonine | 3.44E+04 | 2.82E+04 | 1.187478 | -0.28816 |
| beta-Cubebene | 4.99E+04 | 4.08E+04 | 1.693615 | -0.28993 |
| L-Methionine | 1.51E+07 | 1.23E+07 | 1.149658 | -0.291 |
| alpha-Terpinene | 2.33E+05 | 1.91E+05 | 2.095654 | -0.29103 |
| Uridine | 5.25E+05 | 4.28E+05 | 1.016832 | -0.29612 |
| alpha-Cadinene | 4.71E+04 | 3.82E+04 | 1.433231 | -0.30145 |
| Lysopc 18:3 | 7.75E+07 | 6.29E+07 | 1.42908 | -0.30208 |
| Punicic acid | 1.90E+07 | 1.54E+07 | 2.207745 | -0.30522 |
| 1,4-Xylene | 1.23E+05 | 9.92E+04 | 1.655366 | -0.31383 |
| N-Alpha-Acetyl-L-Asparagine | 3.27E+05 | 2.61E+05 | 1.099239 | -0.32559 |
| Putrescine | 1.02E+06 | 8.15E+05 | 1.305891 | -0.33026 |
| H-Homoarg-Oh | 5.09E+05 | 4.03E+05 | 1.169421 | -0.33672 |
| N-Acetyl-L-Glutamic Acid | 3.34E+05 | 2.64E+05 | 1.358271 | -0.33951 |
| Vanillylmandelic acid | 1.88E+04 | 1.48E+04 | 1.389335 | -0.34338 |
| Lysopc 18:0 | 1.66E+08 | 1.30E+08 | 1.494181 | -0.34699 |
| EPA [5Z,8Z,11Z,14Z,17Z-eicosapentaenoic acid] | 1.21E+05 | 9.45E+04 | 1.314612 | -0.3584 |
| PAF C-16 | 1.86E+08 | 1.45E+08 | 1.527785 | -0.35902 |
| Lysope 18:0 | 2.98E+05 | 2.32E+05 | 1.827983 | -0.36079 |
| Lysope 16:0 | 2.11E+06 | 1.64E+06 | 1.637844 | -0.36211 |
| N-Acetyl-L-Leucine | 6.69E+04 | 5.19E+04 | 1.042237 | -0.36649 |
| Acetyl-N-formyl-5-methoxykynurenamine | 1.91E+04 | 1.48E+04 | 1.576757 | -0.36941 |
| Butylparaben | 3.80E+03 | 2.92E+03 | 1.168834 | -0.3781 |
| Hexadecanedioic acid | 1.55E+06 | 1.18E+06 | 1.266111 | -0.39021 |
| L-Glutamine | 3.02E+07 | 2.30E+07 | 1.266845 | -0.39467 |
| 1,7-Dimethylxanthine | 2.47E+04 | 1.88E+04 | 1.188681 | -0.39483 |
| 2-Aminoadipic acid | 5.86E+05 | 4.44E+05 | 1.075617 | -0.39792 |
| 4-Pyridoxic acid | 7.27E+05 | 5.51E+05 | 1.605077 | -0.39898 |
| L-Thyroxine | 2.25E+06 | 1.71E+06 | 1.388274 | -0.39916 |
| Palmitaldehyde | 7.30E+07 | 5.53E+07 | 2.0192 | -0.40033 |
| Lysopg 18:1 | 6.39E+04 | 4.79E+04 | 1.936903 | -0.41615 |
| Lysopc 17:0 | 1.58E+07 | 1.18E+07 | 1.451064 | -0.42276 |
| Arachidic Acid(C20:0) | 2.49E+06 | 1.85E+06 | 1.490743 | -0.42546 |
| N-(2-Methylbenzoyl)glycine | 7.32E+05 | 5.44E+05 | 1.456776 | -0.42843 |
| L-Lysine | 3.19E+07 | 2.37E+07 | 1.333241 | -0.42866 |
| 2,6-Diaminooimelic acid | 6.57E+04 | 4.87E+04 | 1.394087 | -0.43223 |
| 4-Hydroxybenzyl alcohol | 4.45E+04 | 3.29E+04 | 1.200365 | -0.43518 |
| N-Acetylvaline | 3.60E+04 | 2.66E+04 | 1.232979 | -0.43645 |
| Pyrophosphate | 1.68E+04 | 1.24E+04 | 1.077835 | -0.4386 |
| L-Histidine | 8.17E+05 | 6.02E+05 | 1.449924 | -0.44066 |
| 2-Aminoethanesulfonic acid | 7.47E+06 | 5.50E+06 | 1.652791 | -0.44384 |
| 10-UNDECENOIC ACID | 9.65E+05 | 7.07E+05 | 1.039385 | -0.4491 |
| Lysope 18:1 | 4.27E+06 | 3.12E+06 | 1.536338 | -0.45139 |
| 6β-hydroxytestosterone | 3.36E+03 | 2.44E+03 | 1.780071 | -0.4629 |
| Lysopa 16:0 | 4.69E+05 | 3.37E+05 | 1.764925 | -0.47547 |
| Phenylacetyl-L-Glutamine | 7.39E+05 | 5.30E+05 | 1.123705 | -0.47834 |
| (±)12-HETE [(±)12-hydroxy-5Z,8Z,10E,14Z-eicosatetraenoic acid] | 1.25E+04 | 8.94E+03 | 1.484999 | -0.48858 |
| Allantoin | 2.74E+06 | 1.94E+06 | 1.503166 | -0.49832 |
| 5,6-Dihydro-5-Methyluracil | 3.99E+05 | 2.81E+05 | 1.535608 | -0.5085 |
| 12-Hete | 4.93E+03 | 3.45E+03 | 1.318322 | -0.5125 |
| 2'-Deoxyuridine | 8.23E+04 | 5.77E+04 | 1.414076 | -0.51268 |
| Pyrrole-2-Carboxylic acid | 1.37E+04 | 9.57E+03 | 1.334082 | -0.51755 |
| Gamma-Glu-Leu | 1.49E+05 | 1.04E+05 | 1.400333 | -0.52261 |
| D-Glucoronic acid | 7.04E+04 | 4.88E+04 | 1.30771 | -0.52874 |
| Cholesterol | 3.55E+06 | 2.42E+06 | 1.745772 | -0.54855 |
| MARMESIN | 1.22E+05 | 8.21E+04 | 1.229898 | -0.57193 |
| P–Hydroxyphenyl acetic acid | 2.33E+05 | 1.56E+05 | 1.201309 | -0.58143 |
| Nicotinamide-N-Oxide | 2.73E+04 | 1.81E+04 | 1.181027 | -0.59519 |
| L-Cystathionine | 7.20E+04 | 4.72E+04 | 1.002783 | -0.61059 |
| 18-Hydroxycorticosterone | 1.55E+06 | 1.01E+06 | 2.143382 | -0.62177 |
| Cis-11,14,17-Eicosatrienoic Acid (C20:3) | 1.50E+05 | 9.73E+04 | 1.965601 | -0.62204 |
| Xylose | 3.40E+04 | 2.19E+04 | 1.296078 | -0.63071 |
| 2'-Deoxycytidine-5'-Monophosphate | 1.83E+04 | 1.18E+04 | 1.059532 | -0.63198 |
| Guanidinoethyl sulfonate | 3.76E+04 | 2.43E+04 | 1.501354 | -0.63262 |
| Vitamin D3 | 3.89E+04 | 2.50E+04 | 1.564556 | -0.63712 |
| Glutaric acid | 2.68E+06 | 1.72E+06 | 1.350647 | -0.63848 |
| 9-Hpode | 3.39E+04 | 2.17E+04 | 1.776946 | -0.63995 |
| Lysops 22:6 | 1.35E+04 | 8.65E+03 | 1.75727 | -0.64479 |
| tryptophan betaine | 6.75E+06 | 4.30E+06 | 1.159217 | -0.64895 |
| 2-Acetylfuran | 3.01E+07 | 1.91E+07 | 2.067583 | -0.65302 |
| O-Phospho-L-Serine | 3.25E+04 | 2.06E+04 | 1.276675 | -0.65461 |
| Lysopa 18:0 | 7.23E+04 | 4.58E+04 | 2.008691 | -0.65827 |
| Hyodeoxycholic acid | 1.04E+05 | 6.59E+04 | 2.00019 | -0.66011 |
| D-Arabinose | 3.14E+04 | 1.97E+04 | 1.563776 | -0.67356 |
| Mandelic acid | 4.72E+05 | 2.96E+05 | 1.319491 | -0.67438 |
| 2-Methylsuccinic acid | 2.73E+06 | 1.71E+06 | 1.367482 | -0.6769 |
| Ureidoisobutyric acid | 4.39E+04 | 2.74E+04 | 1.108466 | -0.67919 |
| 3-Hydroxyanthranilic acid | 4.03E+04 | 2.51E+04 | 1.256758 | -0.68448 |
| Taurochenodesoxycholic acid | 1.20E+05 | 7.42E+04 | 1.948025 | -0.69398 |
| N6-Succinyl adenosine | 8.08E+05 | 4.97E+05 | 1.976834 | -0.70092 |
| Ubiquinone-1 | 4.19E+04 | 2.57E+04 | 1.569812 | -0.70857 |
| Indoleacrylic acid | 2.63E+05 | 1.60E+05 | 1.203985 | -0.71749 |
| Isoquinoline | 2.25E+05 | 1.30E+05 | 1.161196 | -0.78646 |
| Linoleic Acid (C18:2N6C) | 2.15E+06 | 1.24E+06 | 1.806432 | -0.79798 |
| INDOLE-3-CARBINOL | 2.19E+05 | 1.25E+05 | 1.209384 | -0.80452 |
| Dodecanoic acid(C12:0) | 2.70E+05 | 1.52E+05 | 2.117741 | -0.82617 |
| L-Homocitrulline | 3.42E+05 | 1.92E+05 | 1.784011 | -0.83521 |
| Ethylmalonate | 1.88E+05 | 1.05E+05 | 1.356575 | -0.83666 |
| Indole-3-Carboxaldehyde | 1.32E+05 | 7.37E+04 | 1.170778 | -0.83745 |
| 11-Cis-retinol | 6.14E+04 | 3.39E+04 | 2.012543 | -0.85858 |
| Α-Linolenic acid(C18:3N3) | 1.61E+04 | 8.81E+03 | 1.588182 | -0.86919 |
| γ-Linolenic acid(C18:3N6) | 1.34E+04 | 7.23E+03 | 1.700771 | -0.88685 |
| 7-ketodeoxycholic acid | 4.81E+03 | 2.57E+03 | 1.145319 | -0.9046 |
| Palmitoleic acid (C16:1) | 1.06E+06 | 5.22E+05 | 1.939649 | -1.01812 |
| N-Oleoyl glycine | 8.15E+03 | 3.77E+03 | 2.052196 | -1.11271 |
| 13-HOTrE [13S-hydroxy-9Z,11E,15Z-octadecatrienoic acid] | 4.02E+04 | 1.68E+04 | 2.061182 | -1.25905 |
| Uridine 5'-Diphospho-N-Acetylgalactosamine | 1.92E+04 | 7.77E+03 | 2.098032 | -1.30178 |
| 8,15-Dihete | 7.53E+04 | 2.83E+04 | 1.910542 | -1.41012 |
| Chenodeoxycholic acid | 2.94E+06 | 9.79E+05 | 1.364163 | -1.58879 |

^1^VIP, variable importance in the projection; FC, fold change.

**
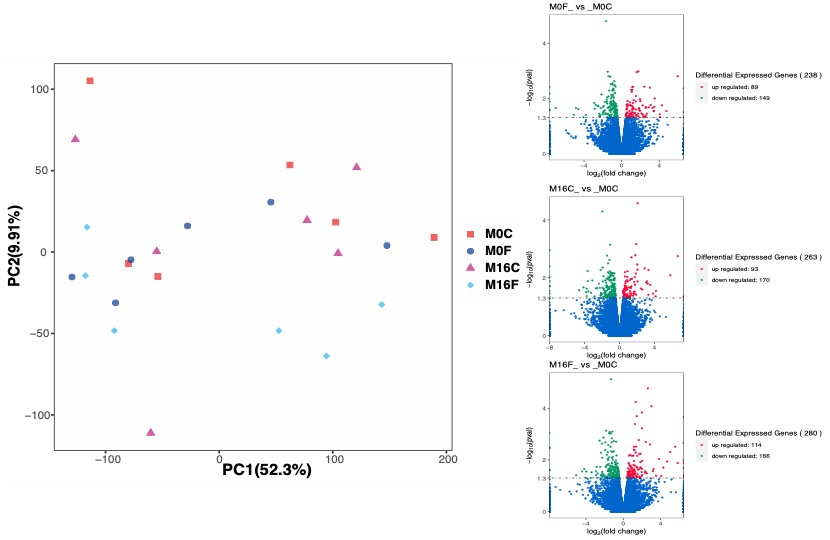
**

**Figure S1.** The principal components analysis of the four groups and Volcano plot analyses of total genes in the liver of the three comparisons (M0C vs. M0F, M0C vs. M16C, M0C vs. M16F).

**
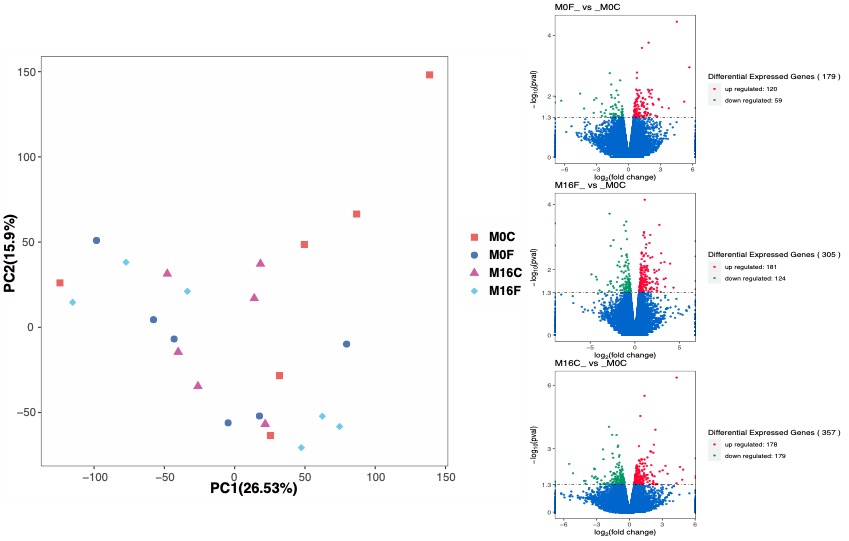
**

**Figure S2.** The principal components analysis of the four groups and Volcano plot analyses of total genes in the muscle of the three comparisons (M0C vs. M0F, M0C vs. M16C, M0C vs. M16F).
